# Second Generation Inactivated Eastern Equine Encephalitis Virus Vaccine Candidates Protect Mice against a Lethal Aerosol Challenge

**DOI:** 10.1371/journal.pone.0104708

**Published:** 2014-08-12

**Authors:** Shelley P. Honnold, Russell R. Bakken, Diana Fisher, Cathleen M. Lind, Jeffrey W. Cohen, Lori T. Eccleston, Kevin B. Spurgers, Radha K. Maheshwari, Pamela J. Glass

**Affiliations:** 1 Virology Division, United States Army Medical Research Institute of Infectious Diseases, Fort Detrick, MD, United States of America; 2 Department of Pathology, Uniformed Services University of the Health Sciences, Bethesda, MD, United States of America; Georgia State University, United States of America

## Abstract

Currently, there are no FDA-licensed vaccines or therapeutics for eastern equine encephalitis virus (EEEV) for human use. We recently developed several methods to inactivate CVEV1219, a chimeric live-attenuated eastern equine encephalitis virus (EEEV). Dosage and schedule studies were conducted to evaluate the immunogenicity and protective efficacy of three potential second-generation inactivated EEEV (iEEEV) vaccine candidates in mice: formalin-inactivated CVEV1219 (fCVEV1219), INA-inactivated CVEV1219 (iCVEV1219) and gamma-irradiated CVEV1219 (gCVEV1219). Both fCVEV1219 and gCVEV1219 provided partial to complete protection against an aerosol challenge when administered by different routes and schedules at various doses, while iCVEV1219 was unable to provide substantial protection against an aerosol challenge by any route, dose, or schedule tested. When evaluating antibody responses, neutralizing antibody, not virus specific IgG or IgA, was the best correlate of protection. The results of these studies suggest that both fCVEV1219 and gCVEV1219 should be evaluated further and considered for advancement as potential second-generation inactivated vaccine candidates for EEEV.

## Introduction

Eastern equine encephalitis virus (EEEV), an arbovirus, is an important human and veterinary pathogen belonging to one of seven antigenic complexes in the genus *Alphavirus*, family *Togaviridae*. EEEV is considered the most deadly of the mosquito-borne alphaviruses due to the high case fatality rate associated with clinical infections, reaching as high as 75% in humans and 90% in horses [1]. In patients that survive, the neurologic sequelae are often severe and debilitating. Although natural infections are acquired by mosquito bite, EEEV is also highly infectious by aerosol, making it a potential agent of bioterrorism.

There are four antigenic subtypes of EEEV, one that circulates in North America and the Caribbean (NA EEEV), and three that circulate in Central and South America (SA EEEV). The strains differ in their geographic, epidemiologic, pathogenic, phylogenetic, and evolutionary characteristics and recently, Arrigo et al. [2] proposed that the SA EEEV variants be classified as a distinct species called *Madariaga virus* (MADV). NA EEEV strains are highly conserved, monophyletic, and temporally related, while SA EEEV strains are highly divergent, polyphyletic, co-circulating, and geographically associated [2]. NA EEEV results in approximately 5–8 human cases yearly, often with devastating outcomes, while SA EEEV historically has had little to no association with human disease, despite evidence of human exposure in endemic areas [3]. However, in a recent outbreak in Panama, 8 patients were hospitalized with eastern equine encephalitis (EEE) as a result of SA EEEV infection [4]. NA EEEV is also listed as a category B agent by the National Institute of Allergy and Infectious Diseases (NIAID) due to its virulence, its potential use as a biological weapon, and the lack of a licensed vaccine or effective antiviral treatment for human infections.

**Figure 1:**
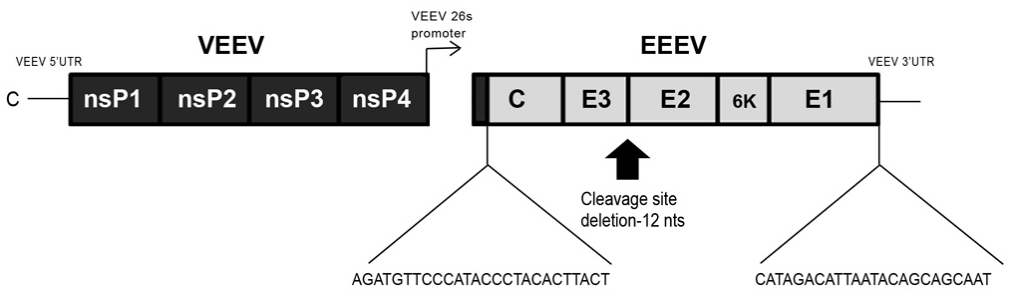
Schematic of the pCVEV1219 plasmid. The 5′- and 3′-UTR, nonstructural proteins, 26S and a portion of the capsid protein was derived from the V3000 cDNA clone. The remainder of the capsid protein through the E1 glycoprotein stop codon was derived from the EEEV FL91-4679 cDNA clone. In addition, the site for furin cleavage was deleted; therefore, furin is unable to cleave E2 and E3 and they are transported to the cell surface as their precursor (PE2).

CVEV1219 is a genetically modified strain of EEEV (Figure 1), containing the nonstructural proteins of Venezuelan equine encephalitis virus (VEEV) and the structural proteins of EEEV. Additionally, the furin cleavage site within the PE2 glycoprotein is deleted, which significantly attenuates the virus *in vitro*. During cellular processing of the wild-type virus, furin, a cellular protease, cleaves E2 and E3, E3 is then released and E1 and E2 form a heterodimer which is transported to the cell surface [6]. In the mutant virus, the site for cleavage is deleted; therefore, furin is unable to cleave E2 and E3 and they are transported to the cell surface as their precursor (PE2), resulting in a change in the surface structure such that there is an extra surface projection. This is a lethal mutation; however, rescued virus contains compensatory mutations which alter the glycoprotein interactions and resuscitate the virus [6], [7]. This mutant virus is similar to V3526, the furin cleavage deletion mutant of VEEV. The PE2 domain of V3526 has been shown to be immunogenic given that monoclonal antibodies directed to this domain were able to protect mice from lethal VEEV challenge [8]. Additionally, this vaccine candidate showed great promise in animal studies and protected against multiple serotypes of VEEV, while circumventing the vaccine interference that is often observed with alphaviruses [7], [9]. However; modified live vaccines are not without problems, as was recently seen when V3526 was tested in phase I clinical trials. V3526 protected mice from both subcutaneous and aerosol challenge [7]. Additionally, V3526 provided protection within one week of vaccination and protection persisted for at least one year against both homologous and heterologous VEEV [9]. Nonetheless, when it was transitioned to phase 1 human clinical trials it induced unacceptable side effects and was not further pursued (Parker MD, personal communication).

Inactivating an attenuated-live virus provides an additional layer of safety in the formulation of the vaccine candidate. Since there is no virus replication during immunization with inactivated vaccines, the virus cannot revert to virulence, as can occur with modified-live vaccines. However, the currently available IND EEEV vaccine, PE6, is a substandard vaccine. This inactivated vaccine produces neutralizing titers that do not protect against aerosol challenge in animal models, as seen in this study. In humans, there are often a significant number of non-responders and individuals must be administered booster vaccinations routinely to maintain an adequate titer [10]. Additionally, the IND EEEV vaccine, as produced, cannot be licensed because the manufacturing procedures do not meet current GMP practices. Therefore, we optimized processes to inactivate a genetically modified strain of EEEV using formalin, INA, and gamma-irradiation, since all three of these methods were successful in inactivating V3526 and most induced significant immune responses and were at least partially protective against a subcutaneous or aerosol challenge [11]–[14].

Formalin has historically been used in inactivated vaccines licensed by the FDA. Although it induces cross-linking of proteins, which could affect epitope immunogenicity, it has recently been used to successfully inactivate both V3526 [15] and Japanese encephalitis virus [16]. Another chemical compound recently used to inactivate enveloped viruses is 1,5-iodonaphthylazide (INA), which is a hydrophobic photo-reactive probe that binds to transmembrane anchors of proteins upon photo-activation with UV light [17]. Traditionally, it has been used for labeling membrane proteins and evaluating their dynamics and fusion as well as for studying protein-membrane interactions [18]. However, with far-UV irradiation (310–360 nm), INA alkylates the transmembrane domains of viral proteins, resulting in their inactivation, while maintaining the integrity of the external domains. INA is unique in that it preserves membrane protein structural integrity and therefore is potentially useful for vaccine applications. INA has recently been used inactivate V3000 (a full-length cDNA clone derived from the virulent Trinidad donkey strain of VEEV) [12], V3526 [11], HIV [19], SIV [19], influenza virus [20], and Ebola virus [21]. Gamma-irradiation has also been used experimentally to inactivate enveloped viruses. Gamma-irradiation inactivates viruses by generating strand-breaks in the genetic material, with little impact on the antigenic structure and biological integrity of proteins and has been used successfully to inactivate V3526 [13] and influenza A virus [22]–[24].

The objectives of this study were to evaluate the ability of formalin, INA, and gamma-irradiation to inactivate a genetically modified strain of EEEV, CVEV1219, and to evaluate the immunogenicity and protective efficacy of these inactivated EEEV (iEEEV) vaccine candidates in BALB/c mice using various routes, doses and schedules. The protective efficacy of the immunological responses was evaluated by aerosol challenge with EEEV strain FL93-939. While all three methods completely inactivated CVEV1219, the formalin and gamma-inactivated formulations of CVEV1219 provided 100% protection against an aerosol exposure when administered by different routes and schedules at various doses.

## Materials and Methods

### CVEV1219

The CVEV1219 virus was a generous gift of Dr. Michael Parker. CVEV1219 is a chimeric virus rescued from the pCVEV1219 plasmid. The pCVEV1219 plasmid contains the VEEV 5′- and 3′-untranslated region (UTR), the VEEV nonstructural proteins through the 26S promoter along with the 5′ end of the capsid protein, all derived from the full-length cDNA clone of VEEV, V3000 [6]. The remainder of the capsid protein, through the E1 stop codon, was derived from a clone of EEEV FL91-4679 (Figure 1). This virus lacks the furin cleavage site (nucleotides 8552–8566, AGGAGAACCAGGAGA) and E2 His167 (nucleotides 9065–9067, CAT). It also contains a G to T transversion at nucleotide position 9021 resulting in an Arg to Leu change at E2-152 and a T to A transversion at nucleotide position 11342 within the untranslated region. These changes resuscitated the lethal cleavage deletion mutation, presumably by altering the glycoprotein interactions. The resulting virus contained trimers of PE2-E1 heterodimers.

### Formalin inactivation

Sucrose purified CVEV1219 virus stock aliquots with known viral titer and protein concentration were suspended in 1X Dulbecco’s PBS (DPBS) (GIBCO, Invitrogen Corp., Grand Island, NY) at a protein concentration of 100 µg/ml. One milliliter aliquots of virus, in cryovial tubes, were treated with 37% Formaldehyde solution stabilized with 10% methanol (Thomas Scientific, Swedesboro, NJ) at a final concentration of 0.1% and 25% Buminate, human serum albumin (Baxter Healthcare Corp., Westlake Village, CA) at a final concentration of 0.5%. Samples were incubated for 18 hours at 37°C in a Forma Scientific orbital shaker at 200 rpm (ThermoFisher Scientific, Waltham, MA). Formalin was removed by pelleting the virus through a 20% sucrose cushion at 40,000 rpm (273865 RCF) for four hours using a SW 41Ti rotor and a Beckman, L7 ultracentrifuge (Beckman Coulter, Inc., Brea, CA). The formalin treated CVEV1219 (fCVEV1219) pellet was suspended in 250–500 µL of 1X DPBS overnight at 4°C. Aliquots were combined and protein concentration was determined using a BCA Protein Assay kit (Thermo Scientific, Rockford, IL) as per manufacturer’s instructions.

### INA inactivation

INA inactivation was performed as previously described with some modification [12]. Sucrose purified CVEV1219 virus stock was suspended in 1X DPBS at a protein concentration of 500 µg/ml in a clear transparent tube, and from this point on, reduced lighting conditions were used. INA (Biotium, Hayward, CA) was added to the virus suspension to a final concentration of 200 µM and then samples were incubated for 20 min in the dark at room temperature (RT). Following incubation, the samples were centrifuged at 1000 rpm for 1 min, supernatant was transferred to new tube and glutathione was added to a final concentration of 20 mM. The virus suspension was irradiated using a BLAK-Ray^R^ B-100 series longwave ultraviolet lamp with a 100 watt bulb (UVP, Upland, CA). Samples were irradiated for a total of 10 min. Thereafter full light conditions were used and aliquots were stored at −80°C until used for *in vitro* and *in vivo* testing.

### Gamma-irradiation

Sucrose purified CVEV1219 virus stock aliquots with known viral titer and protein concentration were frozen at −80°C and were irradiated with 8–10 Mrad (80,000–100,000 Gy) of gamma-irradiation in a 484R AECL gammacell cobalt irradiator (J.L. Shepherd and Assoc., San Fernando, CO). Samples were aliquoted and stored at −80°C until used for *in vitro* and *in vivo* testing.

### Testing for residual infectivity *in vitro* by serial passage

Inactivated virus preparations were tested for residual infectivity by five serial passes of 72–96 hours each on baby hamster kidney (BHK-21) cells at a MOI of at least 1000. One hundred microliters of the diluted vaccine candidate was added to one well of a 6 well plate (Costar) containing BHK-21 cells at 50% confluency. Samples were tested in duplicate. Plates were incubated for 1 hour at 37°C, 5% CO_2_ with humidity. One to two milliliters of supplemented EMEM was added to each well and plates were incubated for 3–4 days at 37°C, 5% CO_2_ with humidity (pass 1). After incubation, wells were evaluated for the presence of cytopathology. If no cytopathic effect (CPE) was observed 200 µL of supernatant was transferred to new plates containing BHK-21 cells at 50% confluency and the procedure was replicated. This procedure was repeated for a total of 5 passes. Supernatant from the 5th pass was collected and stored at −80°C for further *in vitro* testing by standard plaque assay and indirect immunofluroescent analysis.

### Testing for residual infectivity *in vitro* by standard plaque assay

A standard plaque assay on Vero cell monolayers was used to determine if any infectious particles remained in each of the inactivated virus preparations. Supernatant from the 5th pass on BHK-21 cells was collected and added undiluted, in duplicate, to 6-well plates containing a confluent monolayer of Vero cells. Plates were incubated at 37°C for 1 hour. Following the incubation period, wells were overlaid with 0.5% agarose in supplemented EBME media, and plates were incubated at 37°C at 5% CO_2_ for 24 hr. Thereafter, cells were stained by the addition of a second agarose overlay prepared as above containing 5% neutral red. The plates were incubated at 37°C at 5% CO_2_ for 24 hr. Residual infectivity was quantitated by counting defined plaques (neutral red exclusion areas).

### Testing for residual infectivity *in vitro* by immunofluorescent assay

Indirect immunofluorescent assay was also used to determine if any infectious particles remained in each of the inactivated virus preparations. Briefly, 100 µL of supernatant from the 5th pass on BHK-21 cells was added in duplicate to a chamber of a Lab-Tek 8-well chamber slide system (Nalge Nunc International, Rochester, NY) containing confluent BHK-21 cells. Slides were incubated for 1 hour at 37°C, 5% CO_2_ with humidity and then 300 µL of supplemented EMEM was added. Slides were incubated overnight at 37°C, 5% CO_2_ with humidity. The following day, the chamber was removed and the slide was rinsed in DPBS and air dried at RT. Cells were fixed in ice cold acetone for 10 min at RT and then were air dried. To visualize viral proteins, slides were incubated with EEEV hyperimmune mouse ascites fluid, EEE-HMAF, in 50% glycerol diluted 1∶500 in PBS with 5% FBS for 1 hour at RT in a humidified chamber. Slides were then rinsed in DPBS and incubated with the secondary antibody (FITC-labeled goat anti-mouse antibody diluted 1∶80 in DPBS with 5% FBS) in the dark for 30 min at RT in a humidified chamber. Slides were then rinsed in DPBS and coverslip mounted using Vectashield mounting medium for fluorescence with DAPI (Vector Laboratories, Inc., Burlingame, CA). Slides were evaluated using a Nikon Eclipse E800 fluorescent microscope.

### Testing for residual infectivity *in vivo*

Inactivated virus preparations were tested *in vivo* by intracranial inoculation of suckling mice. Specific pathogen free late-term pregnant female BALB/c mice (NCI, Frederick, MD) were housed in an animal biosafety level 3 (ABSL-3) facility in cages equipped with microisolators and were provided food and water *ad libitum* throughout the study. The room temperature was 23±1°C and periods of light and dark were maintained on a 12 h cycle. Newborn suckling mice were allowed to acclimate for 1 day prior to inoculation. Suckling mice were inoculated by the intracranial route (IC) with 10 µL of inactivated CVEV1219 or sterile saline (negative control) using a 50 or 100 µL Hamilton syringe with a 22–26 gauge needle. The mice were observed twice daily for 14 days for clinical signs of illness, cannibalization, and death. Suckling mice that were severely ill or moribund were euthanized. The brains from surviving mice were homogenized and ten microliters of the supernatant was then injected IC into a second group of suckling mice. These mice were observed twice daily for 14 days for clinical signs of illness or cannibalization. The brain from any suckling mouse that succumbed to infection or was euthanized due to illness was homogenized and frozen for viral titer analysis.

### IND EEEV vaccine

Control mice received the investigational new drug (IND) EEEV vaccine, formulated from the PE6 WRAIR strain of EEEV prepared in chick embryo tissue (The Salk Institute, Government Services Division, Swiftwater, PA). This vaccine was prepared by inactivation with formaldehyde and neutralized with sodium bisulfite. Neomycin sulfate equivalent to 50 µg/ml, neomycin base, and 0.25% human serum albumin were added. The vaccine was dried and stored at −20°C until use. For use, the vaccine was reconstituted with 3 ml of sterile water and mice were given 0.5 ml subcutaneously (4 µg) per manufacturer’s instructions.

### Vaccination of mice

Specific pathogen free 6–8-week-old female BALB/c mice (NCI, Frederick, MD) were selected for these studies because they are one of the most widely used inbred strains used in animal experimentation, immunology, and virus research. These animals were housed in cages equipped with microisolators and were provided food and water *ad libitum* throughout the study. The room temperature was 23±1°C and periods of light and dark were maintained on a 12 h cycle. Mice were acclimated for 1 week before vaccination. Mice were observed daily and weighed every other day for 14 d post-vaccination. Three weeks after vaccination, retro-orbital sinus blood collection was performed under Isoflurane anesthesia (Webster Veterinary, Devens, MA) using the IMPAC^6^ (VetEquip, Pleasanton, CA). One to two days later, vaginal flushes were obtained as previously described [25]. Mice were restrained and douched with 100 µL of PBS. Both serum and vaginal flush samples were individually collected and stored at −80°C for further analysis.

For the portions of the study involving challenge of mice with wild type EEEV, mice were housed in an animal biosafety level 3 (ABSL-3) facility. Mean weight of mice prior to challenge was 20.17 g (range 18.42 to 21.45 g). Humane endpoints were used during all mouse studies. All research was conducted under a U.S. Army Medical Research Institute of Infectious Diseases IACUC approved protocol in compliance with the Animal Welfare Act, PHS Policy, and other Federal statutes and regulations relating to animals and experiments involving animals. The facility where this research was conducted is accredited by the Association for Assessment and Accreditation of Laboratory Animal Care, International and adheres to principles stated in the 8^th^ Edition of the Guide for the Care and Use of Laboratory Animals, National Research Council, 2011. Sample size for efficacy experiments was determined based on the use of a one-tailed Fisher’s exact test to compare survival rates with adequate (>80%) power at a 95% confidence level.

In the first study, mice were vaccinated intranasally (IN), intramuscularly (IM) or subcutaneously (SC) with 0.1–5 µg of inactivated EEEV (iEEEV) vaccine candidate: CVEV1219 inactivated by formalin (fCVEV1219), INA (iCVEV1219), or gamma-irradiation (gCVEV1219) or sterile saline as described in Table 1. One group of control mice received the investigational new drug (IND) EEEV vaccine, following the manufacturer’s instructions (4 µg per dose, on d0 and d28, subcutaneously). In the second study, mice were vaccinated with 3 µg intranasally (IN), 5 µg subcutaneously (SC), or 1 µg intramuscularly (IM) with fCVEV1219 as described in Table 2. For intranasal vaccinations, iEEEV vaccine candidates were diluted to the appropriate concentration such that each dose was given in a total of 20 µL, 10 µL per nostril, using a pipet under isoflurane anesthesia. For intramuscular and subcutaneous vaccinations, iEEEV vaccine candidates were diluted to the appropriate concentration such that each dose was given in a total of 100 µL in the rear leg or 200 µL interscapular, respectively.

**Table 1:** Vaccine study design evaluating iEEEV candidates.

Group	Dose	Route	Vaccination Schedule	Bleed, VF Schedule	Aerosol Challenge	# Mice
1	5 µg iEEEV	IM, SC, IN	D0	D21	D28	10
2	3 µg iEEEV	IM, SC, IN	D0	D21	D28	10
3	1 µg iEEEV	IM, SC, IN	D0	D21	D28	10
4	0.1 µg iEEEV	IM, SC, IN	D0	D21	D28	10
5	Sterile Saline	IM, SC, IN	D0	D21	D28	10
6	5 µg iEEEV	IM, SC, IN	D0, D28	D21, D49	D56	10
7	3 µg iEEEV	IM, SC, IN	D0, D28	D21, D49	D56	10
8	1 µg iEEEV	IM, SC, IN	D0, D28	D21, D49	D56	10
9	0.1 µg iEEEV	IM, SC, IN	D0, D28	D21, D49	D56	10
10	Sterile Saline	IM, SC, IN	D0, D28	D21, D49	D56	10
11	EEEV IND	SC	D0, D28	D21, D49	D56	10

**Table 2:** Extended vaccine study design evaluating iEEEV candidates.

Group	Vaccine Candidate	Dose	Route	Vaccination Schedule	Blood, VF Collection	Aerosol Challenge	# Mice
1	fCVEV1219	5 µg	SC	D0	D21, D56	D63	10
2	fCVEV1219	3 µg	IN	D0	D21, D56	D63	10
3	fCVEV1219	1 µg	IM	D0	D21, D56	D63	10
4	No Vax	–	–	D0	D21, D56	D63	5
5	fCVEV1219	5 µg	SC	D0, D56	D21, D56, D77	D84	10
6	fCVEV1219	3 µg	IN	D0, D56	D21, D56, D77	D84	10
7	fCVEV1219	1 µg	IM	D0, D56	D21, D56, D77	D84	10
8	No Vax	–	–	D0, D56	D21, D56, D77	D84	5

### Enzyme-linked immunosorbent assay (ELISA)

Serum and vaginal flush antibody responses to the vaccine candidates were evaluated by ELISA as previously described [7], [13], [25]. Briefly, Costar EIA/RIA 96-well high-binding plates (Corning Inc., Corning, NY) were coated with 0.2 µg of sucrose purified EEEV strain FL93-939 per well and incubated overnight, or up to 1 week, at 4°C. The following day, plates were blocked with Dulbecco’s phosphate buffered saline (DPBS) (GIBCO Invitrogen Corp., Grand Island, NY) containing 0.05% Tween 20 (Sigma-Aldrick, St. Louis, MO) and 5% nonfat dry milk (Becton Dickinson and Co., Sparks, MD) (PBSTM) for 2 hours at 37°C. The plates were washed 3 times with PBST using the BioTek ELx405 microplate washer (BioTek Instruments, Inc., Winooski, VT). Mouse sera were diluted in PBSTM containing 1% heat inactivated fetal bovine serum (GIBCO Invitrogen Corp., Grand Island, NY), added to the plate and serially diluted 1∶2 and then incubated for 1–2 hours at 37°C. Plates were washed 3 times with PBST followed by the addition of one of five peroxidase-labeled goat anti-mouse Ig (IgG 1∶50,000; IgG1 1∶50,000; IgG2a 1∶100,000; IgG2b 1∶10,000; IgA 1∶10,000) (Bethyl Laboratories, Inc., Montgomery, TX). The plates were incubated with the secondary antibody for 1 hr at 37°C and then washed 3 times with PBST. The ABST Peroxidase substrate (KPL, Gaithersburg, MD) was added to each well and color developed for approximately 20–30 min at which time the optical density (OD) at 410 nm was determined using a Spectramax M5 microplate reader (Molecular Devices, Sunnyvale, CA). The negative control was pooled serum from unvaccinated adult mice and was diluted 1∶100. The positive control was pooled serum from previously vaccinated adult mice which survived challenge with parental virus and was diluted 1∶400. Endpoint titers were determined as the highest two-fold dilution that produced an OD greater than the mean OD of the negative controls wells plus 3 standard deviations.

### Plaque-reduction neutralization test (PRNT)

Virus-neutralizing antibody responses were titrated as previously described [7]. Briefly, sera were serially diluted two-fold in Hank’s Balanced Salt Solution (HBSS) containing HEPES red (USAMRIID, Fort Detrick, MD) and 2% FBS and incubated overnight with virus. The serum-virus mixture was then added in duplicate to 6-well plates containing a confluent monolayer of Vero cells and the procedure was performed as described for the plaque assay. The endpoint titer was determined to be the highest dilution with an 80% or greater reduction (PRNT 80) of the number of plaques observed in control wells. The assay limit of detection was calculated to be 5 pfu/ml by this method.

### Challenge virus

EEEV strain FL93-939 was obtained from Dr. Scott Weaver, UTMB, Galveston, TX. Sucrose gradient purified aerosol challenge stock was prepared from seed stock (P1) through an additional passage (P2) in Vero cells. Virus titer was determined by standard plaque assay on Vero cell monolayers. Virus was aliquoted and frozen at −70 to −80°C prior to use. Challenge virus was diluted in Eagle’s minimum essential medium (EMEM) (USAMRIID, Fort Detrick, MD).

### Aerosol challenge

Aerosol exposures were conducted in a whole-body bioaerosol exposure system. A Collison nebulizer (BGI, Inc., Waltham, MA) was used to generate small (1 µm mass median aerodynamic diameter) diameter particles for each acute 10 min exposure. Briefly, mice were placed in wire cages, which were then placed into a chamber where they were exposed to aerosolized virus for 10 min. The ‘presented’ dose was estimated by calculating the respiratory minute volume (V_m_) using Guyton’s formula, expressed as Vm = 2.10×W_b_^0.75^ where W_b_ = body weight (gm) based on the average group weights the day of exposure. The presented dose was then calculated by multiplying the estimated total volume (V_t_) of experimental atmosphere inhaled by each animal (V_t_ = V_m_ x length of exposure) by the empirically determined exposure concentration (C_e_) (‘presented dose’ = C_e_ x V_t_). Exposure concentration, expressed in plaque-forming units (PFU)/L, was determined by isokinetic sampling of the chamber with an all-glass impinger (AGI) (Ace Glass, Vineland, NJ). AGI samples were titrated by standard plaque assay on Vero cell monolayers [26]. Back titration of challenge virus preparations were determined by standard plaque assay using Vero cells.

### Statistics

Fisher’s exact tests with stepdown Bonferroni adjustment were used to compare survival rates. Logistic regression of survival by log_10_-transformed immune response factors with backward elimination to select a set of statistically-significant covariates from among the covariates (vaccine candidate, dose, route, and schedule) was used to determine odds ratios. Logistic regression by probit analysis of survival status by immune response factor was used to predict log_10_-transformed immune response factors that would yield a probability of survival of 90% and 99%.

## Results

### CVEV1219 inactivation

**Figure 2:**
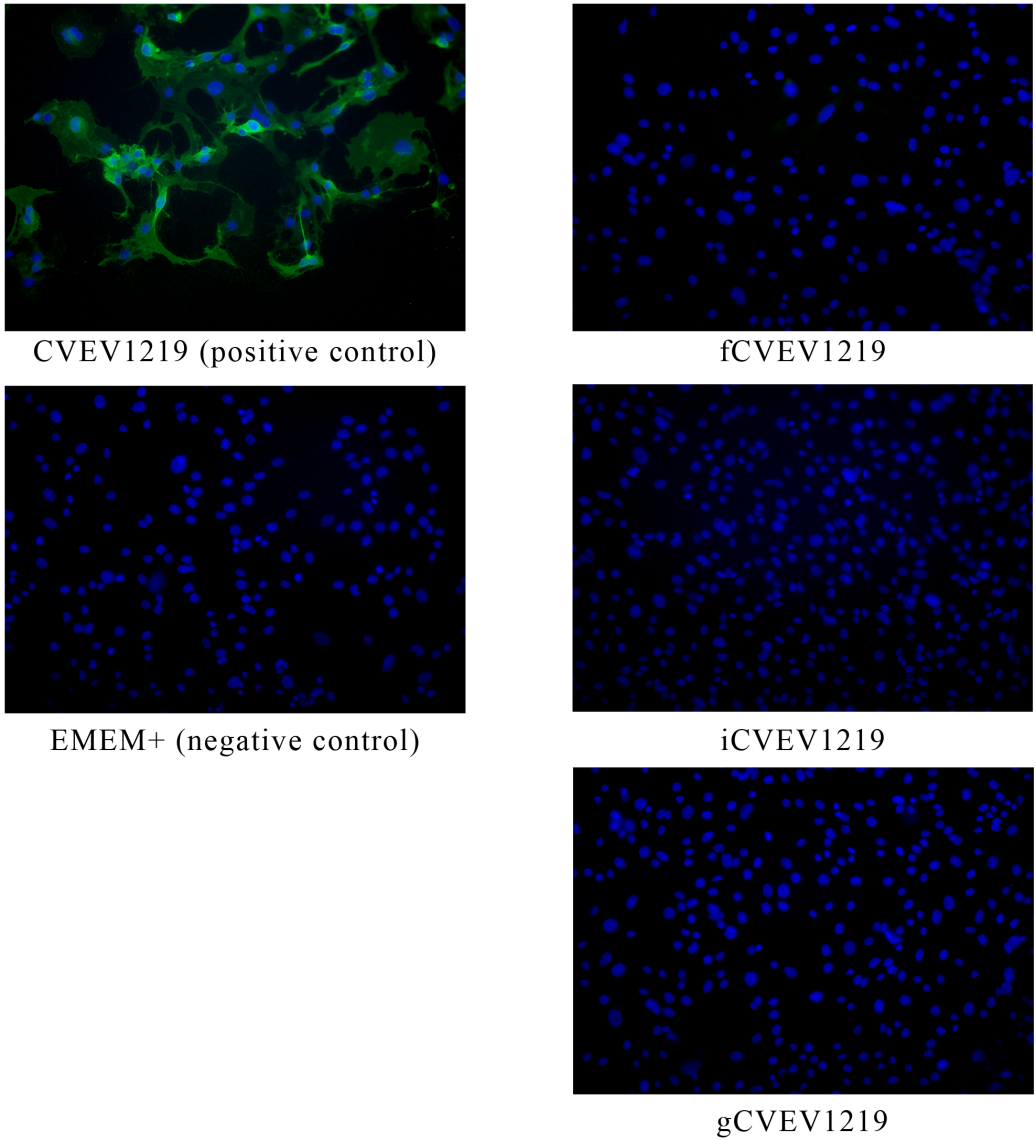
Immunofluorescent detection of EEEV antigen. Inactivated samples were passed five times on BHK-21 cells. The supernatant from the 5^th^ pass was evaluated by immunofluorescent assay using a polyclonal EEEV Ab to ensure no residual infectivity.

CVEV1219 was completely and consistently inactivated when treated with 0.1% formalin after an 18 hour incubation period at 37°C with shaking. After purification through a 20% sucrose cushion, 75–80% of the starting protein concentration was recovered as determined by the BCA method. Higher concentrations of CVEV1219 (500 µg/ml) were completely and consistently inactivated using 200 µM of INA combined with 10 min of UV exposure. The highest concentrations (800–1000 µg/ml) of CVEV1219 were completely and consistently inactivated with exposure to 10 Mrad, when inactivated in bulk quantities of 20–30 ml. An aliquot from all inactivated samples was tested for residual infectivity *in vitro* by serial passage in BHK-21 cells with no detection of CPE. The supernatant from the 5th pass was tested for residual infectivity using the standard plaque assay and immunofluorescent assay (IFA). No virus was detected using the standard plaque assay (data not shown) and no viral antigen was detected by IFA (Figure 2).

After all methods of inactivation (formalin, INA, gamma-irradiation) were optimized (Table 3), sufficiently large quantities of CVEV1219 were inactivated by each method and tested for residual infectivity *in vitro* using the approach described above. These samples were then tested for residual infectivity *in vivo*. Although the *in vitro* assessment of viral inactivation is sensitive, intracranial inoculation of suckling mice is a more sensitive indicator and considered the “gold-standard” for assessing inactivation/attenuation of alphaviruses [11], [27]–[29].

**Table 3:** Optimized Inactivation Conditions for CVEV1219.

	Virus (µg/ml)	Method of Inactivation
Formalin(fCVEV1219)	100	0.1% formalin → incubate 18 hr37°C with shaking → purify through a20% sucrose cushion
INA(iCVEV1219)	500	200 µM INA → incubate in the darkfor 20 min at RT → 10 min UV, mix every 2 min
Gamma-irradiation(gCVEV1219)	939	Sample frozen → 10 Mrad (100,000 Gy)in a cobalt irradiator

Interestingly, while the formalin-inactivated CVEV1219 (fCVEV1219) and the INA-inactivated CVEV1219 (iCVEV1219) passed both the *in vitro* and *in vivo* testing for residual infectivity, the gamma-irradiated CVEV1219 (gCVEV1219) that received 8 Mrad passed all of the *in vitro* testing; however, 2/12 suckling mice showed clinical signs of disease and died or were euthanizied after intracranial inoculation (Table 4). The brains from these mice were homogenized and virus was detected by standard plaque assay in the brain of one mouse. No other mice in this group showed any clinical signs of disease. A new preparation of CVEV1219 was gamma-irradiated with 10 Mrad and these samples passed both the *in vitro* and *in vivo* testing for residual infectivity (Table 4).

**Table 4:** In Vivo Inactivation Evaluation in Suckling Mice.

	Pass #1		Pass #2	
Groups	% Survival	# died/total	% Survival	# died/total
fCVEV1219	100	0/13	100	0/11
iCVEV1219	100	0/11	100	0/13
gCVEV1219 (8 Mrad)	83.3	2/12*		
gCVEV1219 (10 Mrad)	100	0/11	100	0/16
PBS	100	0/8	100	0/8
CVEV1219	0	5/5	0	7/7

*One mouse succumbed and virus was not detected in the brain by plaque assay. The other mouse showed clinical signs of disease and was euthanized; virus was present in the brain by plaque assay.

### Clinical observations

In the first study mice were vaccinated and challenged as described in Table 1. While all groups lost a small amount of weight, less than 2%, 1 day post-vaccination, all groups quickly recovered and weighed more than their original weight by 3 days post-infection (dpi) (data not shown).

Almost animals that were vaccinated and survived, regardless of vaccine candidate, dose, or route, did not exhibit clinical signs of disease following aerosol challenge. However, animals that were vaccinated but were not protected against an aerosol challenge, clinical signs of disease and weight loss began as early as 2–3 dpi (IM group), similar to saline controls, or slightly later, at 3–4 dpi (IN group), or had a wider range of disease onset, 2–5 dpi (SC group) regardless of vaccine candidate, dose, or schedule. Overall, the majority of animals in which clinical signs of disease were observed succumbed to infection or were euthanized by 7 dpi; however, a small percentage of animals in which minimal clinical signs, such as ruffled fur, were observed made a full recovery. Onset of clinical disease in saline control mice in each group was between 2–4 dpi, while the EEEV IND vaccine control mice showed signs of disease at 3 dpi. One animal in this group became sick, but recovered (data not shown).

### Intranasal vaccination efficacy

**Figure 3:**
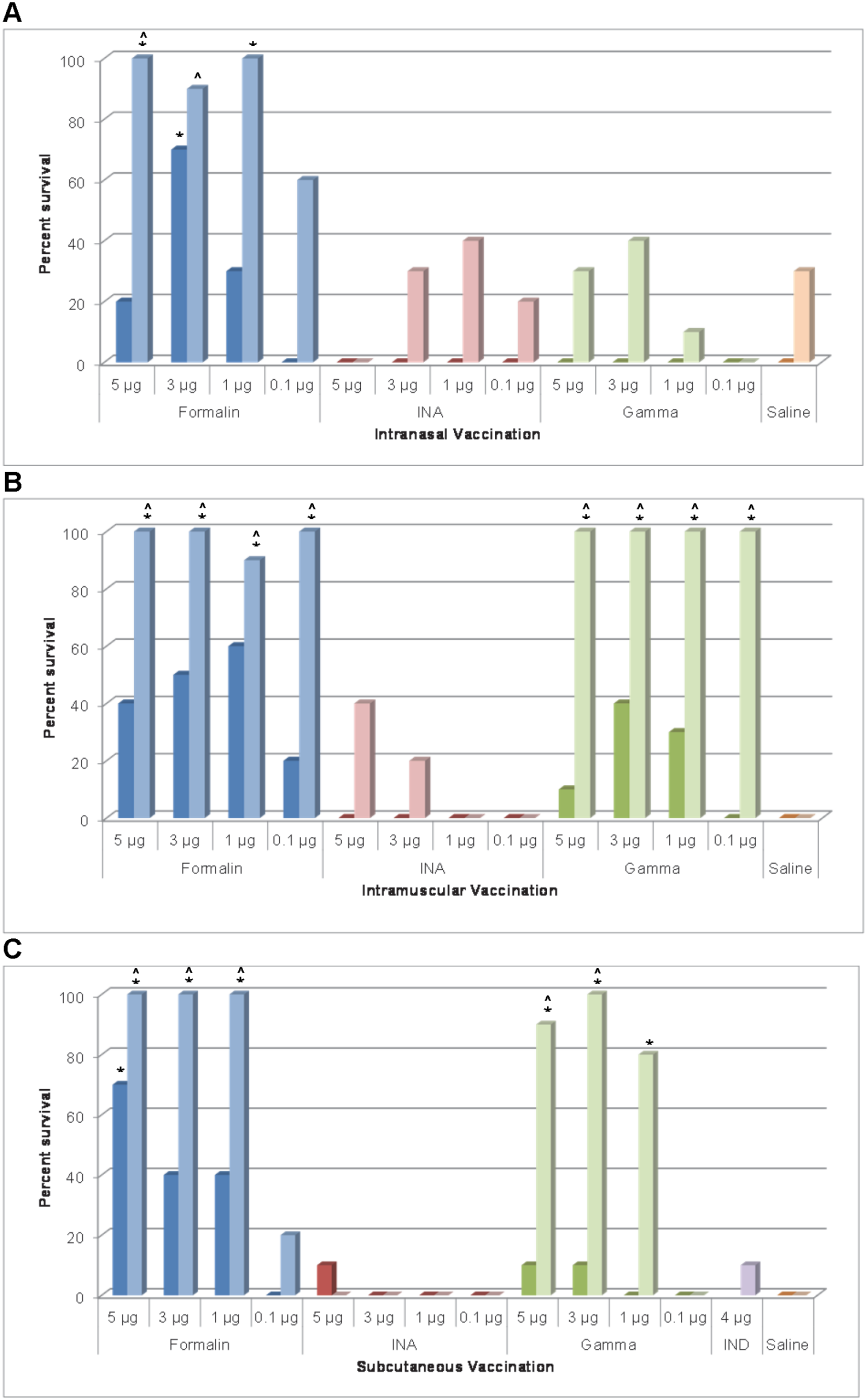
Protective efficacy of iEEEV vaccine candidates. Groups of BALB/c mice (n = 10) were administered one (dark bars) or two doses (light bars) of iEEEV vaccine candidate at doses ranging from 5–0.1 µg by IN, IM, or SC routes. Mice were challenged by aerosol with at least 100LD_50_ of EEEV strain FL93-939, 28 days after the final vaccination, and were monitored for 28 days for mortality and clinical signs of disease (*p-value<0.05 for pairwise comparison to saline group; ∧p-value<0.05 for pairwise comparison to IND vaccine group).

In mice given a single IN vaccination, only fCVEV1219 provided statistically significant partial protection against aerosol challenge at the 3 µg dose (70%) (p = 0.031), while both iCVEV1219 and gCVEV1219 provided no protection regardless of dose (Figure 3A, dark bars). In mice given two IN vaccinations, fCVEV1219 provided 90–100% protection against an aerosol challenge at the 1 µg dose or higher. However, both iCVEV1219 and gCVEV1219 did not provide significant partial protection at any dose (Figure 3A, light bars). For unknown reasons, 3/10 control mice challenged on d56 survived aerosol challenge; however, fCVEV1219 groups which were completely protected (5 µg and 1 µg doses) were still statistically significant (p = 0.031). Additionally, statistically significant differences in survival rates were noted between mice that received one vaccination and mice that received two vaccinations of fCVEV1219 IN at the 5, 1, and 0.1 µg doses (p<0.05). When comparing the IN vaccination regime to the standard EEEV IND regime (2 doses, 4 µg per dose, SC), the mice given fCVEV1219 in a 2 dose regime at 5, 3, or 1 µg dose had statistically higher survival rates than the mice that received the EEEV IND vaccine (p<0.05).

### Intramuscular vaccination efficacy

In mice given a single IM vaccination, both fCVEV1219 and gCVEV1219 provided partial protection (30–60%) against aerosol challenge at multiple doses, but the level of protection was not statistically significant (Figure 3B, dark bars). However, both fCVEV1219 and gCVEV1219 provided 100% protection against aerosol challenge at multiple doses when given in a two dose regimen (p<0.001) (Figure 3B, light bars). iCVEV1219 was unable to provide significant protection in either the one or two dose regimens. Statistically significant differences in survival rates were noted between mice that received 1 vaccination and mice that received two vaccinations of either fCVEV1219 at the 5, 3, or 0.1 µg doses or gCVEV1219 at the 5, 3, 1, and 0.1 µg doses (p<0.05). When comparing the IM vaccination regimen to the standard EEEV IND regime (2 doses, 4 µg per dose, SC), the mice given fCVEV1219 or gCVEV1219 in a two dose regimen at all doses had statistically higher survival rates than the mice that received the EEEV IND vaccine (p<0.05).

### Subcutaneous vaccination efficacy

In mice given a single SC vaccination, only fCVEV1219 at the highest dose (5 µg) provided significant partial protection against an aerosol challenge (p = 0.031) when compared to saline controls (Figure 3C, dark bars), while all other doses of fCVEV1219 and all doses of iCVEV1219 and gCVEV1219 did not. However, both fCVEV1219 and gCVEV1219 provided 100% protection at multiple doses when given in a two dose regime (p = 0.001) (Figure 3C, light bars). Statistically significant differences in survival rates were also noted between those mice that received 1 vaccination and mice that received two vaccinations of either fCVEV1219 at the 1 and 3 µg doses or gCVEV1219 at the 5, 3, and 1 µg doses (p<0.01). When comparing the SC vaccination regime to the standard EEEV IND regime (2 doses, 4 µg per dose, SC), the mice given fCVEV1219 at the 5, 3, or 1 µg dose or gCVEV1219 at the 5 and 3 µg dose in a 2 dose regime had statistically higher survival rates than the mice that received the EEEV IND vaccine (p<0.05).

### Systemic immune response to vaccination

**Figure 4:**
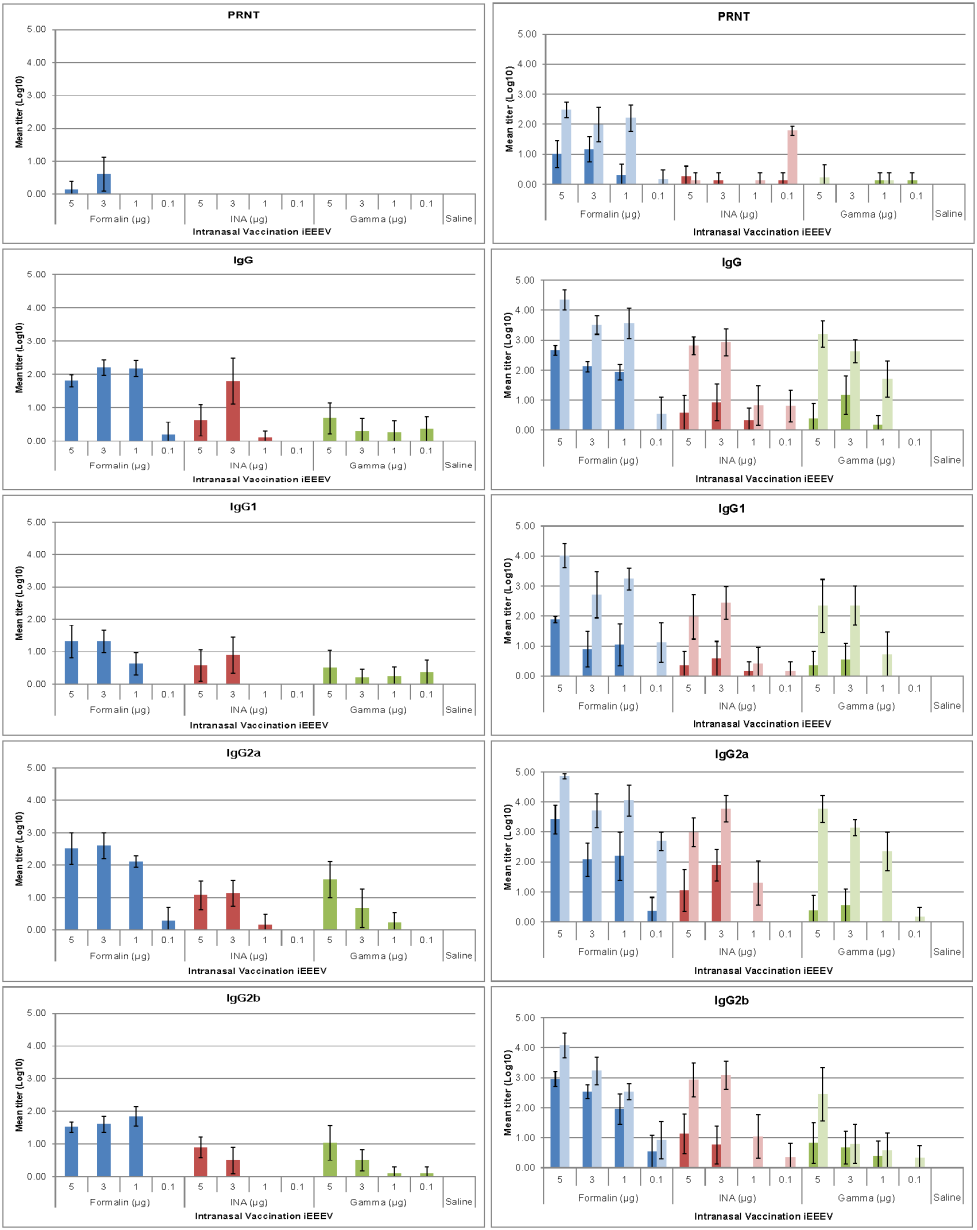
Serum antibody responses in mice vaccinated intranasally with iEEEV vaccine candidates. Groups of BALB/c mice (n = 10) were vaccinated once (graphs on left) or twice (graphs on right) with one of three iEEEV vaccine candidates (formalin-inactivated, INA-inactivated, or gamma irradiated) at doses ranging from 5–0.1 µg by the IN route. Serum was collected 21 d after each vaccination. Neutralizing antibody responses were determined by PRNT and serum antibody levels were determined by ELISA. In all graphs, dark bars represent the mean group titer 21 d after the first vaccination (n = 10); light bars represent the mean group titer 21 d after the second vaccination (n = 10). Standard error bars represent 2 times the SE of the mean (SE = SDxsqrt(n)).

**Figure 5:**
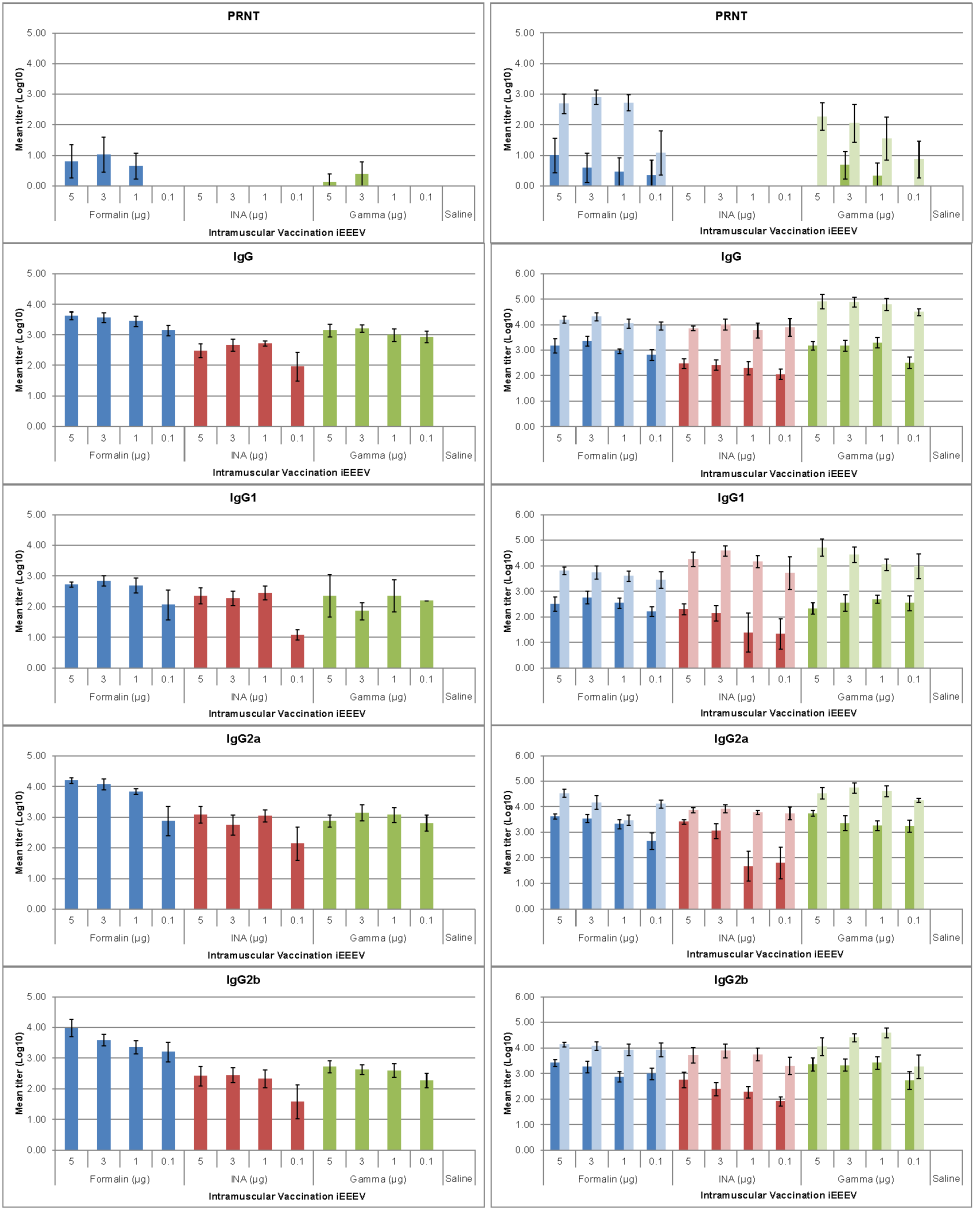
Serum antibody responses in mice vaccinated intramuscularly with iEEEV vaccine candidates. Groups of BALB/c mice (n = 10) were vaccinated once (graphs on left) or twice (graphs on right) with one of three iEEEV vaccine candidates (formalin-inactivated, INA-inactivated, or gamma irradiated) at doses ranging from 5–0.1 µg by the IM route. Serum was collected 21 d after each vaccination. Neutralizing antibody responses were determined by PRNT and serum antibody levels were determined by ELISA. In all graphs, dark bars represent the mean group titer 21 d after the first vaccination (n = 10); light bars represent the mean group titer 21 d after the second vaccination (n = 10). Standard error bars represent 2 times the SE of the mean (SE = SDxsqrt(n)).

**Figure 6:**
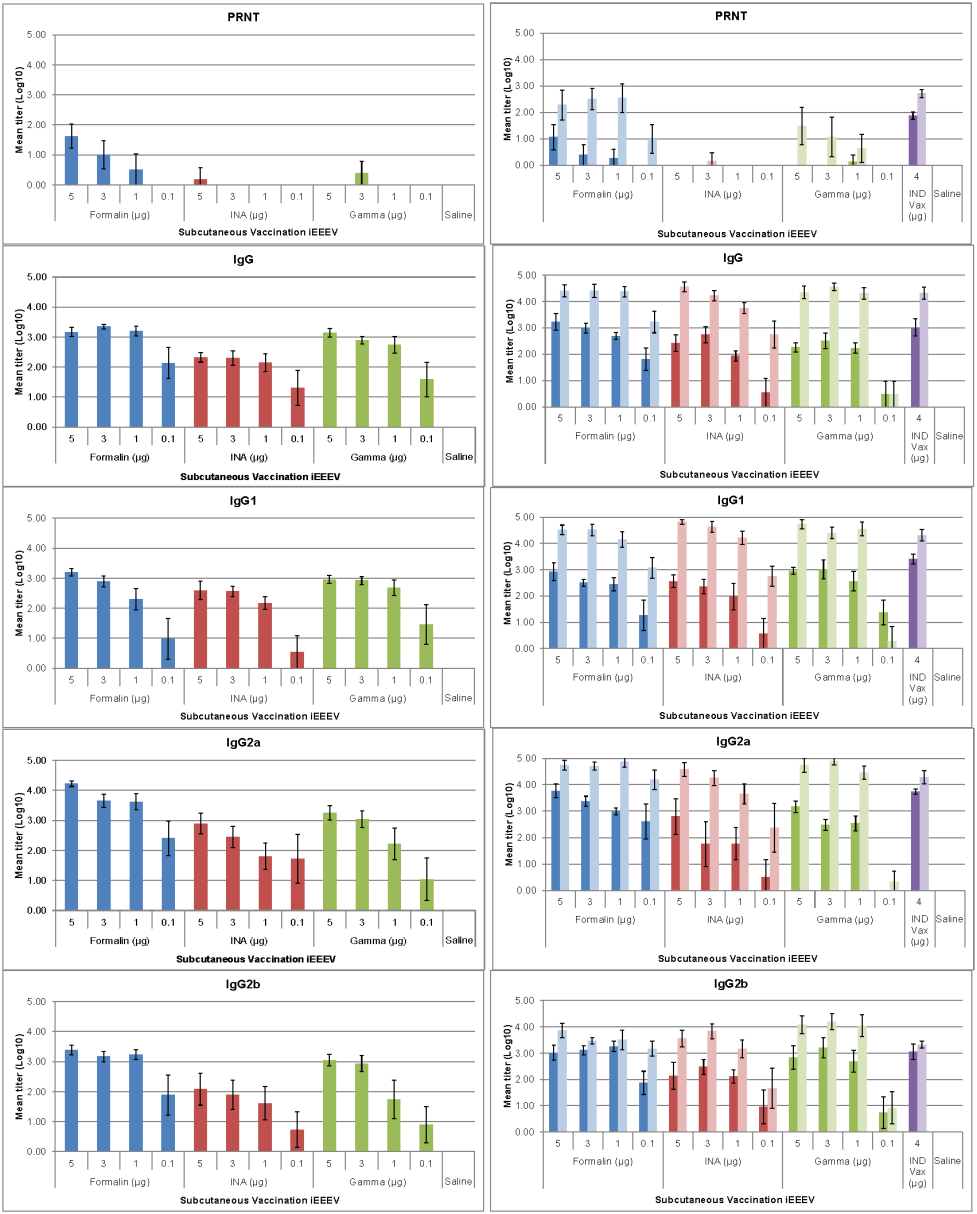
Serum antibody responses in mice vaccinated subcutaneously with iEEEV vaccine candidates. Groups of BALB/c mice (n = 10) were vaccinated once (graphs on left) or twice (graphs on right) with one of three iEEEV vaccine candidates (formalin-inactivated, INA-inactivated, or gamma irradiated) at doses ranging from 5–0.1 µg by the SC route. Serum was collected 21 d after each vaccination. Neutralizing antibody responses were determined by PRNT and serum antibody levels were determined by ELISA. In all graphs, dark bars represent the mean group titer 21 d after the first vaccination (n = 10); light bars represent the mean group titer 21 d after the second vaccination (n = 10). Standard error bars represent 2 times the SE of the mean (SE = SDxsqrt(n)).

Serum neutralizing antibody responses as well as all immunoglobulins measured were greater after the second vaccination regardless of vaccine candidate, dose or method of inactivation (Figures 4–6). When evaluating antibody response, serum neutralizing antibody, not virus specific IgG or IgA, were the best correlate of protection.

Logistic regression was utilized to assess whether there was a significant increase in odds of survival for each unit increase in immune response factor. Table 5 contains the overall odds ratio for the odds of survival for each unit increase in immune response factor, the 95% confidence limits for the odds ratio, and p-value for each immune response parameter. For example, taking into account all methods of inactivation, routes of inoculation, and doses, for every unit increase (a ten-fold increase in titer) in serum neutralizing antibody immune response, as measured by the PRNT assay after two vaccinations (at day 56), an animal would have over a 4-fold increase in odds of surviving an aerosol exposure; whereas, in the same animals, an increase in each unit of IgG, IgG2a or IgG2b would expect to increase the odds of survival by approximately two-fold. According to statistical analysis, regardless of dose, route, or inactivation method, a log_10_-transformed PRNT value of 1.57 after one vaccination or 2.20 after 2 vaccinations would protect 90% of mice from an aerosol challenge against EEEV strain FL93-939, while much higher titers of IgG, IgG1, IgG2a or IgG2b would be needed to protect the same number of animals (Table 6). This analysis was also done to evaluate the various methods of inactivation as well as the route of inoculation and the vaccine regimen.

**Table 5:** Odds ratios for the odds of survival for each unit increase in immune response factor.

Immune ResponseParameter	Day*	Odds Ratio	OR 95% CI	p-value
IgG	21	1.099	(0.797, 1.515)	0.5667
	56	2.344	(1.489, 3.690)	**0.0002**
IgG1	21	1.784	(1.310, 2.430)	**0.0002**
	56	1.807	(1.236, 2.642)	**0.0023**
IgG2a	21	1.567	(1.189, 2.064)	**0.0014**
	56	2.013	(1.245, 3.254)	**0.0043**
IgG2b	21	2.247	(1.636, 3.087)	**<0.0001**
	56	2.094	(1.441, 3.043)	**0.0001**
PRNT	21	1.881	(1.240, 2.853)	**0.0029**
	56	4.249	(2.464, 7.326)	**<0.0001**
VF IgA	21	1.344	(0.829, 2.179)	0.2305
	56	1.474	(0.891, 2.438)	0.1309

*Table represents data from all groups for day 21, and only for animals receiving two vaccinations for day 56.

**Table 6:** Predicted titers required for 90% and 99% survival following standard vaccination schedule.

		IgG		IgG1		IgG2a		IgG2b		PRNT	
		90%	99%	90%	99%	90%	99%	90%	99%	90%	99%
**Overall** *	21	5.43	8.13	5.35	8.30	5.64	8.33	4.69	6.77	1.57	2.66
	56	6.19	9.29	7.04	11.34	5.89	8.41	5.65	8.64	2.20	3.65
**Method of Inactivation**											
Formalin	21	5.83	10.50	4.43	8.09	6.63	11.96	5.14	8.80	1.46	2.83
Gamma		5.20	7.83	4.32	6.36	4.83	7.02	4.03	5.59	1.01	1.79
INA		–	–	–	–	–	–	–	–	1.26	1.87
Formalin	56	3.59	6.55	3.32	5.56	4.12	6.03	3.33	6.10	1.77	3.16
Gamma		4.21	5.38	3.92	5.36	4.41	5.39	3.70	5.33	0.85	1.35
INA		–	–	–	–	–	–	–	–	–	–
**Route**											
IN	21	3.47	5.30	2.05	3.16	4.19	6.44	2.94	4.41	0.87	1.38
IM		4.04	5.18	3.03	3.73	4.33	5.53	3.74	4.63	1.01	1.73
SC		4.52	6.11	4.30	5.76	4.81	6.41	3.67	4.47	3.24	5.50
IN	56	6.26	10.17	4.58	7.30	6.60	10.35	5.85	9.74	2.72	4.65
IM		4.10	4.26	6.09	9.72	4.29	4.80	4.24	4.97	1.03	1.66
SC		4.93	5.73	5.98	7.85	4.74	5.01	4.21	4.90	2.68	4.25
Regimen											
1 vaccination	21	5.04	6.52	6.03	8.49	5.40	6.94	5.47	7.38	1.62	2.40
2 vaccinations	56	3.85	5.99	3.88	6.38	4.24	6.62	3.80	5.73	1.37	2.62

*Table represents data from all groups for day 21, and only for animals receiving two vaccinations for day 56.

When evaluating the method of inactivation, it is clear that the gCVEV1219 vaccine candidate required a lower PRNT compared to the fCVEV1219 vaccine candidate to protect the same percentage of animals. No prediction could be made regarding iCVEV1219 due to the decreased efficacy of this vaccine candidate. Additionally, as expected, regardless of whether the animal received one vaccination or two vaccinations, the lowest titers required to protect either 90% or 99% of the animals were the serum neutralizing antibody titers (PRNT), providing additional evidence that a strong neutralizing antibody response is likely to protect most animals against a lethal aerosol challenge.

When comparing the routes of inoculation without regard to dose or method of inactivation, again the lowest titers required to protect either 90% or 99% of the animals were the serum neutralizing antibody titers (PRNT). While the lowest titers required to protect either 90 or 99% of the animals after one vaccination were found in those animals vaccinated by the IN route (0.87 and 1.38, respectively), this did not hold true for those animals receiving two vaccinations, where the lowest titers required to protect 90 or 99% of the animals were found in the animals vaccinated by the IM route (1.03 and 1.66, respectively).

When comparing the vaccination regimen without regard to dose, route, or method of inactivation, again the lowest titers required to protect either 90% or 99% of the animals were the serum neutralizing antibody titers (PRNT). The titers that would yield a probability of survival of 90 or 99% were low and similar between those animals receiving one or two vaccinations. Overall, serum neutralizing antibody responses increased after the second vaccination regardless of vaccine candidate, dose, or method of inactivation, and were the best predictor for survival against an aerosol challenge.

### Mucosal immune response to vaccination

**Figure 7:**
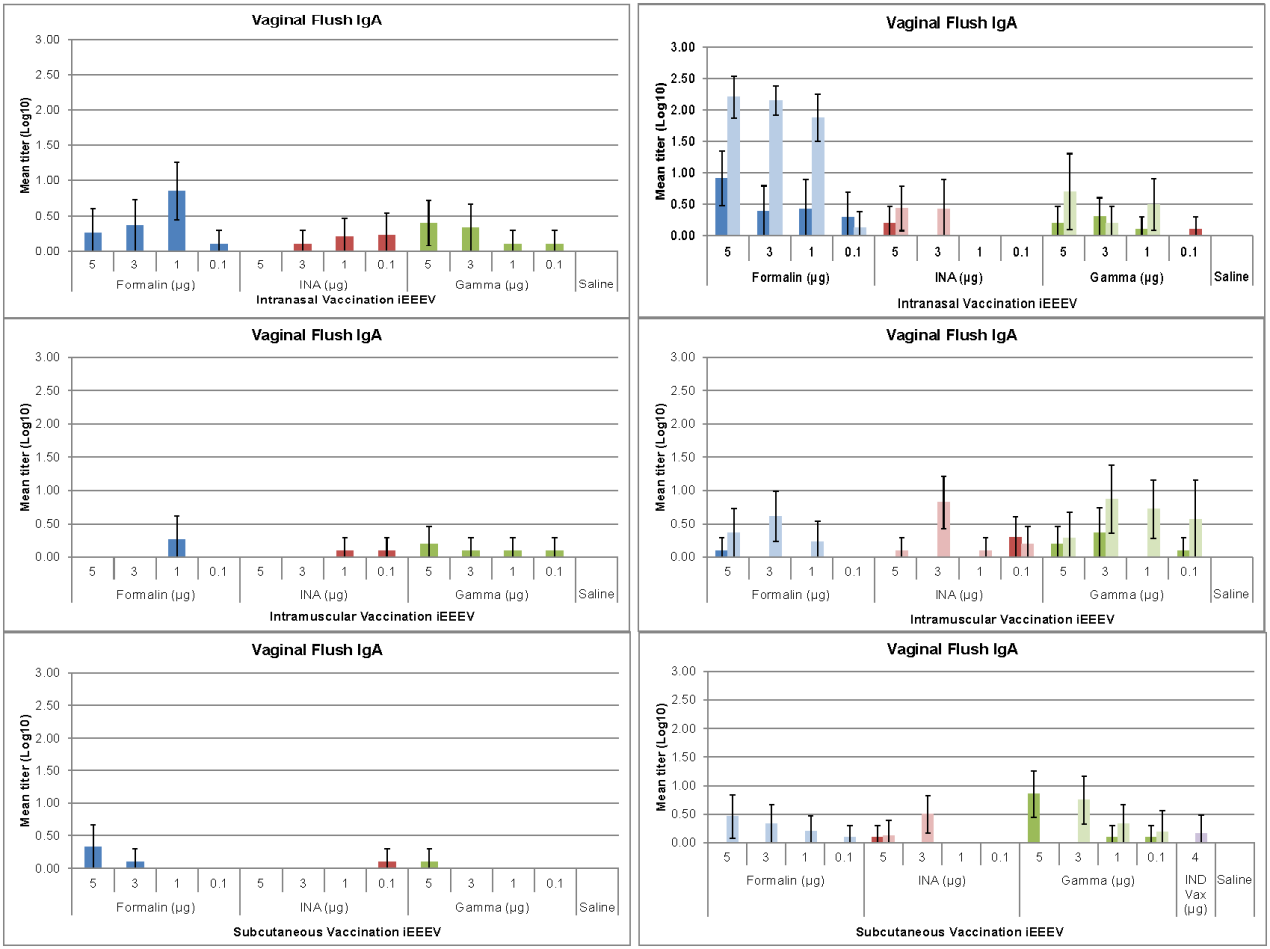
Vaginal flush IgA antibody responses in mice vaccinated with iEEEV vaccine candidates. Groups of BALB/c mice (n = 10) were vaccinated once (graphs on left) or twice (graphs on right) with one of three iEEEV vaccine candidates (formalin-inactivated, INA-inactivated, or gamma irradiated) at doses ranging from 0.1–5 µg by the IN, IM, or SC route. Vaginal flush samples were collected 21 d after each vaccination and virus specific IgA antibody levels were determined by ELISA. In all graphs, dark bars represent the mean group titer 21 days after the first vaccination (n = 10); light bars represent the mean group titer 21 days after the second vaccination (n = 10). Standard error bars represent 2 times the SE of the mean (SE = SDxsqrt(n)).

Vaginal flush (VF) samples were collected from all mice 21 days after each vaccination to assess virus specific mucosal IgA responses. As expected, those mice vaccinated by the IN route had significantly higher IgA levels than those vaccinated by either the IM or SC routes (Figure 7) with increased levels after the second vaccination. However, there was notable variation between samples in all groups, which may have been a result of collection technique and/or cycle differences between mice. Nonetheless, this variability resulted in the odds ratios for both the day 21 and day 56 samples being the lowest with a relatively high p-value (Table 5). For this reason, the predicted log_10_-transformed VF IgA titer that would yield a 90% or 99% probability of survival against an aerosol challenge was not determined.

### Protective efficacy following extended vaccination schedule

Based on the results of the intranasal vaccination study we chose to evaluate the fCVEV1219 vaccine candidate in an extended vaccine regimen and aerosol challenge experiment. These mice were vaccinated and challenged as described in Table 2 and evaluated as in the first experiment.

**Figure 8:**
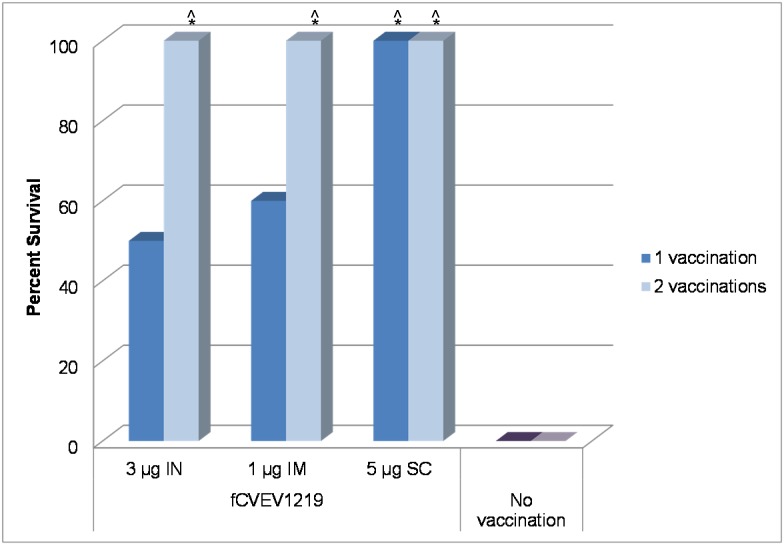
Protective efficacy of fCVEV1219 vaccine candidate when administered on an extended vaccination schedule with aerosol challenge. Groups of BALB/c mice (n = 10) were administered one (dark bars) or two doses (light bars) of fCVEV1219 at doses ranging from 5–1 µg by IN, IM, or SC routes. Half of the mice were challenged by aerosol, with at least 100LD_50_ of EEEV strain FL93-939, 63 days after the first vaccination, while the other half were challenged 28 days after the second vaccination (d84). Mice were monitored for 28 days post-challenge for mortality and clinical signs of disease (*p-value<0.05 for pairwise comparison to control group; ∧p-value<0.05 for pairwise comparison to IND vaccine group from the first study).

Similar to the results of the first study and to unvaccinated controls, those animals that received a single vaccine and were not protected began to lose weight and show clinical signs of disease by 3–4 dpi. All of the animals that showed clinical signs of disease succumbed to infection or were euthanized. In contrast to the results of the first study, 100% of the animals that received a single vaccination of 5 µg fCVEV1219 by the SC route survived when challenge was delayed to 63 days post-vaccination as opposed to 28 days post-vaccination (Figure 8).

All of the animals that received two vaccinations with fCVEV1219 (on day 0 and day 56), regardless of dose and route tested, survived aerosol challenge when challenged 28 days after the second vaccination. Unvaccinated controls showed clinical signs of disease 3 days post-challenge and all succumbed to infection or were euthanized by 5 days post-challenge (Figure 8). As noted in Figure 8, the survival rate was significantly different between the group that received a single SC vaccination (5 µg fCVEV1219) and both the unvaccinated controls and the mice in the first study that received the EEEV IND vaccine (p<0.001). Additionally, statistically significant differences in survival rates were observed between all groups of animals that received 2 vaccinations and both the unvaccinated controls and the mice from the first study that received the EEEV IND vaccine (p<0.0007). A statistically significant difference in the survival rate was noted between mice receiving either one or two vaccinations by the IN route (3 µg fCVEV1219) (p = 0.037).

### Immune response following extended vaccination schedule

**Figure 9:**
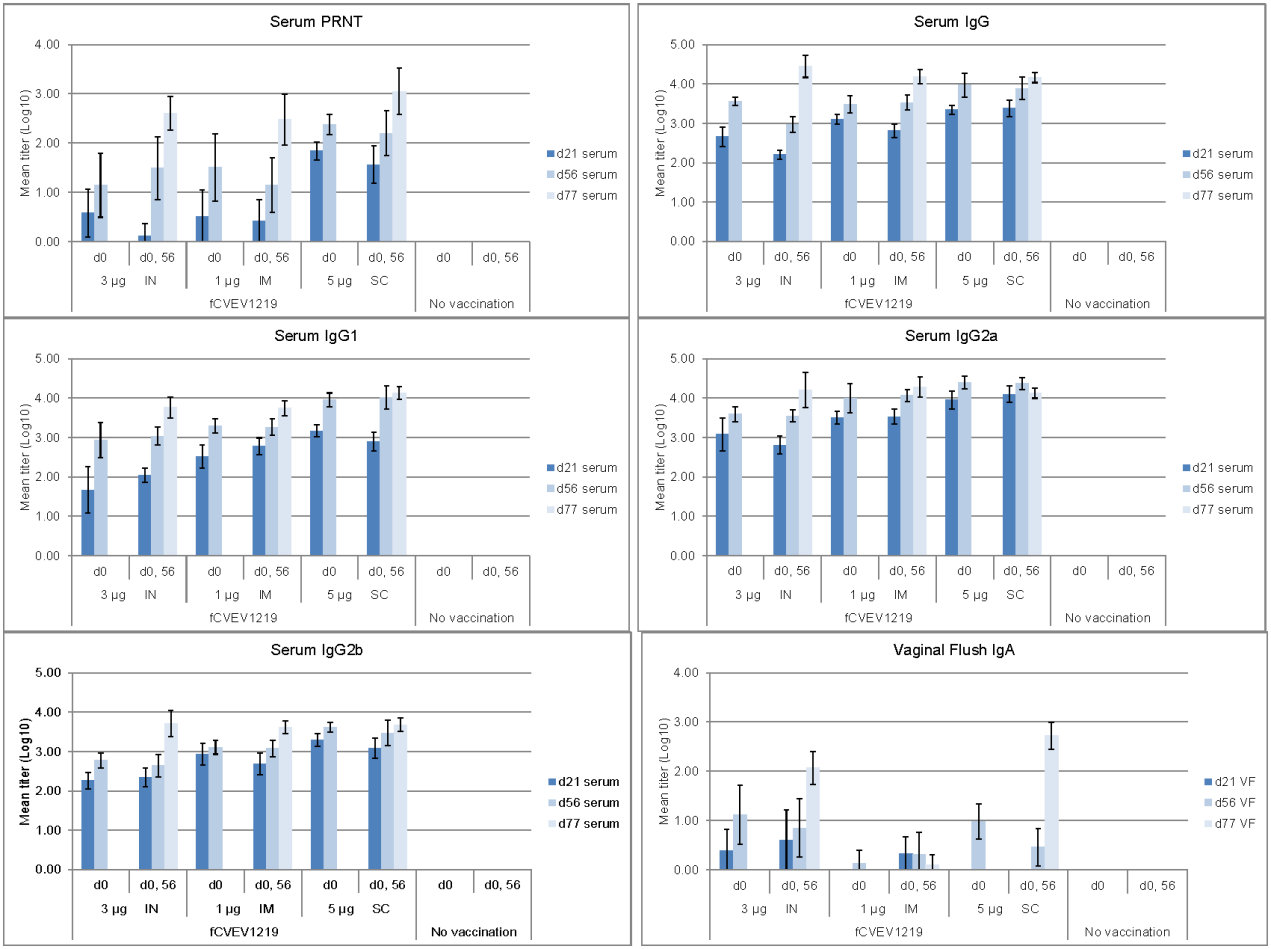
Serum and vaginal flush antibody responses in mice vaccinated with fCVEV1219 vaccine candidate. Groups of BALB/c mice (n = 10) were vaccinated once (d0) or twice (d0, 56) with fCVEV1219 at doses ranging from 5–1 µg by the IN, IM, or SC route. Serum and vaginal flush samples were collected on day 21, 56, and 77 post-vaccination. Neutralizing antibody responses were determined by PRNT and serum and vaginal flush antibody levels were determined by ELISA. Dark bars represent the mean group titer 21 days after the first vaccination (n = 10); medium bars represent the mean group titer 56 days after the first vaccination (n = 10); light bars represent the mean group titer 21 days after the second vaccination (day 77, n = 10). Standard error bars represent 2 times the SE of the mean (SE = SDxsqrt(n)).

For those mice receiving the fCVEV1219 vaccine candidate by any route, both the neutralizing antibody responses as well as all immunoglobulins measured generally increased over time and were the highest after the second vaccination (Figure 9), and levels of serum neutralizing antibody appeared to correlate with survival. The 5 µg SC fCVEV1219 group had the highest titer following the single vaccination. The virus-specific serum IgG levels appeared more similar regardless of dose or route of inoculation. Interestingly, and in contrast to the data in the first vaccine study, the levels of virus-specific IgA in the vaginal flush samples appeared more similar in the groups receiving fCVEV1219 either by the IN or SC routes.

Logistic regression by probit analysis was utilized to assess whether there was a significant increase in odds of survival for each unit increase in immune response factor. However, unlike the results of the first vaccine study, only the odds ratio for IgG1 on day 21 (2.5) and the odds ratio for PRNT on day 56 (3.6) were significant and reproducible (p<0.05) (data not shown). There was insufficient data for the day 77 samples for analysis by logistic regression; therefore, this data set was not analyzed. For all groups, whether they received one or two vaccinations, regardless of dose or route, the levels of virus-specific serum antibody levels of IgG, IgG1, IgG2a and IgG2b were significantly higher in vaccinated mice versus unvaccinated controls at all time points (p<0.0001) (data not shown). The same was true for the PRNT values for all groups at day 56 and day 77 when comparing vaccinated to unvaccinated animals (p<0.05). However, when evaluating the levels of virus-specific IgA in the vaginal flush samples, only those mice receiving either one or two vaccinations by the IN or SC routes had significantly higher levels than the unvaccinated controls (p<0.05) (data not shown). While there were significant differences in the amount of virus specific IgG, IgG1, IgG2a and IgG2b found in those mice receiving one or two vaccinations intranasally at 21 and 56 days post-vaccination (p<0.0007), this was not the case for those mice receiving one or two vaccinations intramuscularly or subcutaneously for any of the immunoglobulins measured at any timepoint (data not shown).

## Discussion

Currently, there are no FDA-licensed vaccines or therapeutics for EEEV for human use. However, there is an investigational new drug (IND) vaccine for EEEV, PE-6, which is currently administered by the U.S. Army Special Immunizations Program to laboratory workers and animal health field workers at risk for exposure to EEEV. This vaccine has several limitations including poor immunogenicity, resulting in the requirement for multiple inoculations; short lived immunity requiring periodic boosters; interference with other alphaviral vaccines; and uncertainty of protective efficacy against an aerosol challenge in animal models [5]. While the goal of vaccine development is to produce a product that closely mimics natural infection; thereby stimulating an appropriate and effective immune response, second generation alphaviral vaccine candidates should utilize current technologies to produce a licensable product that will protect against both natural exposure (subcutaneous) and a potential aerosol exposure (mucosal) to virulent virus, which can be challenging.

Is this study, we optimized processes to inactivate a genetically modified strain of EEEV using formalin, INA, and gamma-irradiation, since all three of these methods were successful in inactivating V3526 and most induced significant immune responses and were at least partially protective against a subcutaneous or aerosol challenge [11]–. CVEV1219 was completely and consistently inactivated by formalin, INA, and gamma-irradiation methodologies. As was shown in this study, it is important to use a multi-system approach, with both *in vitro* and *in vivo* methodologies, to determine residual infectivity and ensure complete and consistent inactivation.

Additionally, we compared the efficacy of iEEEV vaccine candidates (fCVEV1219, iCVEV1219, gCVEV1219) at varying doses, schedules and routes of administration against an aerosol challenge. In the first study, a single-dose administration of the fCVEV1219 vaccine candidate provided partial protection (20–70%) in mice when administered at doses ranging from 1–5 µg by any route, while gCVEV1219 resulted in protection rates ranging from 10–40% when administered IM or SC as a single vaccination at either the 3 or 5 µg doses. However, when mice received two vaccinations 80–100% were protected against an aerosol challenge when vaccinated with fCVEV1219 vaccine candidate by any route or the gCVEV1219 vaccine candidate by the IM or SC routes at doses from 1–5 µg. INA-inactivated CVEV1219 was unable to provide substantial protection against an aerosol challenge by any route, dose or schedule tested. While INA has been used effectively to inactivate several viruses, including VEEV, it has not been previously used in an aerosol challenge model. Therefore, although INA inactivated VEEV can protect mice against a parenteral challenge, it is uncertain whether it would be equally protective against an aerosol challenge [11]. Nonetheless, both fCVEV1219 and gCVEV1219 given in the two dose regimen intramuscularly provided excellent protection (90–100%) against aerosol challenge at all doses.

When evaluating correlates of protection, the above data suggests that the level of serum neutralizing antibodies may be a useful tool in predicting survival and that only a 10-fold increase in titer would increase the odds of survival by more than four fold. However, it should also be noted that those mice that received the EEEV investigational new drug (EEEV IND) at 4 µg SC in a two dose regimen also had similar levels of serum neutralizing antibodies but were not protected from aerosol challenge. This disparity may be due to variation in quality of epitopes following inactivation treatment, the accessibility of epitopes on the cleavage deletion virus, or the immune response generated by each vaccine candidate. It is possible that these second generation vaccine candidates activate the humoral and/or cell-mediated immune system, produce protective non-neutralizing antibody responses as has been noted with Sindbis virus [30], or that antibodies directed against the PE2 (E2 and E3 combined) glycoprotein provide access to additional protective epitopes not recognized when the E3 glycoprotein is cleaved in wild-type virus [8].

Due to the increased protective efficacy seen following two vaccinations of the fCVEV1219 vaccine candidate regardless of route or dose, we investigated whether extending the time between vaccination and challenge would allow for development of a more mature and hence more effective immune response that would increase the protective efficacy of the vaccine following a single dose. While there was no significant change in the protective efficacy when the vaccine was administered intranasally or intramuscularly, there was noteworthy increases in survival when then vaccine was administered subcutaneously, with survival increasing from 40% to 100% following the extended vaccination schedule. And importantly, we achieved 100% protection from an aerosol challenge by all doses and routes evaluated when the vaccine was given in an extended two-dose regimen. As in the first study, the data from this study suggests that the level of serum neutralizing antibody may be a useful tool in predicting survival.

In both studies, vaginal flush virus-specific IgA levels were measured in order to determine if this would be a useful correlate of protection against an aerosol challenge. However, this did not appear to be the case in these studies. As expected, the intranasal route of inoculation typically induced the greatest IgA responses, especially in the two-dose regimen and these animals typically survived aerosol challenge. However, the protective efficacy of fCVEV1219 and gCVEV1219 vaccine candidates administered IM or SC, as a two-dose regimen, were equally high but the vaginal IgA responses were much lower. As noted by the standard error bars, there was significant inter-animal variation, not only in IgA levels, but in the IgG and PRNT levels as well. This variability made group effect determinations difficult.

In this study, the IND EEEV vaccine, which is presently used for at risk personnel, only protected 10% of the mice against aerosol challenge with North American EEEV strain FL93-939. In recent studies, mice vaccinated by the SC route with chimeric Sindbis-EEEV strains or attenuated recombinant EEEV were partially to completely protected against an intraperitoneal challenge with EEEV strain FL93-939 [31], [32]. While these animals were not challenged by the aerosol route, which is typically the most difficult challenge route to protect against [13], a recent study by Roy et al. demonstrated partial protection against aerosol challenge with Sindbis-based vaccine candidates [33]. The Sindbis-based vaccines are partially effective (82% survival) after a single vaccination; however, inactivated vaccines provide an additional level of safety as there is no virus replication. Both formalin-inactivation and gamma-irradiation have been used to inactivate a number of viruses safely and effectively for many years, and formaldehyde is currently used for the FDA licensed polio vaccine. The results of the studies presented in this paper are the first to show that a second-generation inactivated vaccine for EEEV is able to provide 100% protection from an aerosol challenge using different methods of inactivation, doses, routes of inoculation and schedules. The fCVEV1219 vaccine candidate was effective at various doses and routes of inoculation, and provided 100% protection after a single vaccination, making it the most promising candidate in these studies. However, the ultimate goal is to produce a trivalent vaccine that will protect against eastern, western, and Venezuelan equine encephalitis viruses. Current work is ongoing to determine which inactivation method is best for the other encephalitic alphaviruses. Ultimately the results of those studies will assist in down-selection of potential candidates. Future studies will examine the onset, duration, and type of immunity of these second-generation inactivated EEEV vaccines compared to the current IND EEEV vaccine. Additionally, it is possible that the use of adjuvants may be able to boost and/or sustain the immune response, which will be important as these products are moved forward, with the ultimate goal of testing the best candidate in a nonhuman primate model.
